# A role for membrane shape and information processing in cardiac physiology

**DOI:** 10.1007/s00424-014-1575-2

**Published:** 2014-08-17

**Authors:** Ralph Knöll

**Affiliations:** 1 Innovative Medicines and Early Development, Cardiovascular and Metabolic Diseases iMed, AstraZeneca Research and Development Mölndal, Pepparedsleden 1, SE-431 83 Mölndal, Sweden; 2 ICMC (Integrated Cardio Metabolic Centre), Karolinska Institutet, Karolinska University Hospital, Huddinge (M54), SE 141 86 Stockholm, Sweden

**Keywords:** Frank Starling law of the heart, Mechanosensation, Mechanotransduction, Mechanoelectric feedback, Electromechanic feedback, Heart failure, Volume overload, Pressure overload, Pacing-induced heart failure, Force–frequency relationship

## Abstract

While the heart is a dynamic organ and one of its major functions is to provide the organism with sufficient blood supply, the regulatory feedback systems, which allow adaptation to hemodynamic changes, remain not well understood. Our current description of mechanosensation focuses on stretch-sensitive ion channels, cytoskeletal components, structures such as the sarcomeric Z-disc, costameres, caveolae, or the concept of tensegrity, but these models appear incomplete as the remarkable plasticity of the myocardium in response to biomechanical stress and heart rate variations remains unexplained. Signaling activity at membranes depends on their geometric parameters such as surface area and curvature, which links shape to information processing. In the heart, continuous cycles of contraction and relaxation reshape membrane morphology and hence affect cardio-mechanic signaling. This article provides a brief review on current models of mechanosensation and focuses on how signaling, cardiac myocyte dynamics, and membrane shape interact and potentially give rise to a self-organized system that uses shape to sense the extra- and intracellular environment. This novel concept may help to explain how changes in frequency, and thus membrane shape, affect cardiac plasticity. One of the conclusions is that hypertrophy and associated fibrosis, which have been considered as necessary to cope with increased wall stress, can also be seen as part of complex feedback systems which use local membrane inhomogeneity in different cardiac cell types to influence whole organphysiology and which are predicted to fine-tune and thus regulate membrane-mediated signaling.

## Introduction

Heart failure is the leading cause of death worldwide [29]. While we continue to unravel the genetic basis of heart failure and while the equilibrium between cardiomyocyte cell loss and regeneration is severely damaged in heart failure, the functional link between both and how mechanical forces influence these events remains poorly understood [33]. Although the force–frequency relationship, which is an intrinsic property of cardiac muscle resulting in increased force production at higher frequencies, has been described more than a century ago, we are still unable to fully understand this phenomenon (for a recent review: Puglisi et al. [36]). In addition, cardiac hypertrophy and its reverse, cardiac atrophy, are triggered, among others, by an increase or decrease in physical stress, respectively, which are also inherently linked to changes in frequency and hence membrane shape. At the organ level, all of these changes lead to significant remodeling processes, which not only affect cardiac myocytes but also other cell types inherent to the myocardium, the composition of the extracellular matrix (ECM) and of course processes such as angiogenesis [21].

Striated muscle cells such as cardiac myocytes, in contrast to other cell types, produce massive forces and as such are not only sensitive to external but also internal stimuli. It is clear that this force production needs to be fine-tuned and well controlled to avoid dysfunction. This aspect of mechanosensation can be called intracellular mechanosensation in contrast to the well-known perception of mechanical forces from outside the cell. Both effects must be linked, but the underlying molecular mechanisms remain so far unknown [24].

With a molecular mass of up to 4.2 MDa, titin is the largest protein known in biology. It spans from the sarcomeric Z-disc to the M-line and is directly involved in the sensation and transmission of mechanical stimuli, where titin’s I-band region may function as a length sensor (ε = $\frac{l}{\mathrm{l}0}\Big)$, while titin’s Z-disc domain may be involved in the sensation of stress (σ $=\frac{F}{A}\Big)$ [6]. The underlying molecular mechanisms remain poorly defined, but titin interacts at the sarcomeric Z-disc with telethonin or TCAP, which is linked to a striated-muscle-specific, mechanosensitive survival pathway and which can be called mechanoptosis [23]. Mutations in components of the sarcomeric Z-disc are well-known causes of various diseases (i.e., Z-discopathies [18]), including cardiomyopathies [5, 20, 22], and the above-mentioned pathway may well play a role.

An increase in volume (i.e., stretch) is well known to influence the frequency and the regulatory of the beating heart [28], an effect known as mechanoelectrical feedback, and this change in frequency almost certainly will have effects on elastic components of the heart, including titin’s I-band region, and titin’s ability to interact with binding partners [42] and hence will affect its mechanosensory role. In contrast, an increase in pressure most likely primarily affects Z-disc-mediated signaling, an effect which involves Z-disc transcriptional coupling [18, 19]. Truncating titin mutations, which can be found in up to 30 % of dilated cardiomyopathy patients, but also—albeit at a lower frequency of 3 %—in the general population, are thought to be a cause for this type of heart failure [15]. The recently identified molecular mechanism, whereby S-glutathionylation of cryptic cysteins enhances titin elasticity by inhibiting protein folding, may help to understand the underlying pathology and extend our knowledge in regard to effects of missense mutations in this gene. Different signal transduction cascades initiated via titin or the Z-disc may contribute to the development of eccentric and concentric types of hypertrophy observed after volume and pressure overload, respectively. However, our knowledge remains poor in regard to the precise identification of these pathways and how they affect the frequency of the beating heart and hence membrane shape.

### Heart failure, cardiac plasticity, and mechanical forces

At the cellular level, cardiac hypertrophy and atrophy are associated with an increase or decrease in cardiac myocyte (or organ) size, respectively, which (alone) poses a tremendous challenge for every cell. These changes are particularly important for cardiac myocytes not only because new sarcomeres have to be added or removed (positive or negative growth in three dimensions) but also because membrane constituents have to increase or decrease respectively (positive or negative growth in two dimensions). However, due to transcription factor overlap (i.e., there are no specific sets of transcription factors available to separately regulate the transcription of membrane or cellular components), membrane and cellular components have to change proportionately, and a new equilibrium has to be found, which is only possible within certain limits. Therefore, it is no surprise that lethality after myocardial infarction is highest immediately in the days after the incident where remodeling occurs [39], but the feedback mechanisms which link the membrane to remodeling processes remain largely unexplained.

Hypertrophy, within limits and if reversible, might initially be beneficial. However, in the long run (months and years), most likely every hypertrophy is pathological, even in athletes, and no signal transduction pathway is necessarily adaptive or maladaptive, rather the strength and/or the nature of the stimulus determine the outcome (i.e., persistent such as in aortic constriction or intermittent such as during exercise) [21].

Several different but intertwined mechanosensory mechanisms are active in cardiomyocytes (Table 1).

**Table 1:** Summary of current mechanosensory concepts and structures

1. Transmembrane proteins and membrane-associated complexes
(a) mechanosensitive ion channels (i.e., some channels are activated, while others are inactivated by cell stretch) (b) integrins and the dystrophin-associated glycoprotein complex (c) structures: caveolae (d) enzymes: receptor tyrosine kinase (mitogen-activated protein kinase (MAPK))
2. Protein–protein interaction-mediated processes (posttranslational modifications)
3. Sarcomere-related mechanosignaling, including titin (length sensor), actomyosin interaction, AMPK, and Z-disc (tension sensor)

Another way to understand mechanosensation is to introduce tensegrity (Table 2), which describes a concept whereby elements are either under tension (such as the muscles in our body) or in compression (such as the bones) and which has profound consequences for cell biology as it implies that nearly every protein, either directly or indirectly, is involved in processing mechanical information (Table 2). Therefore, naturally occurring mutations or complete loss of these components, such as those observable in genetically altered animals, are expected to affect the perception of biomechanical stress, which in the most cases is true.

**Table 2:** Summary of localized versus tensegrity-based models of signal transduction

1. Localized signal transduction (i.e., the signal is generated in close proximity to the perceived stimulus)
2. Tensegrity (i.e., the stimulus is transmitted before being translated into a biochemical signal)

### Membrane-mediated mechanosensation

The plasma membrane separates the “living” cytoplasm from the outside, serves as a selective barrier, and also integrates the internal cytoplasm with the surroundings by initiating specific signal transduction cascades. By doing so, it allows efficient biochemical signaling primarily via dimensionality reduction in the diffusible space of protein reactants [1]. In addition, the cell membrane not only provides a two-dimensional surface area but also acts as a deformable material whose interaction with the cytoskeleton leads to changes in membrane shapes, a property important for the catalysis of chemical reactions [37]. In cardiac myocytes, the membrane connects to the cytoskeleton (i.e., sarcomeric Z-discs) at structures called costameres, a well-known hot spot for signaling (for an excellent recent review, see Samarel [38]).

According to the law of mass action,

$$ K=\frac{\left[ C\right]\left[ D\right]}{\left[ A\right]\left[ B\right]} $$

the product of the rate of random collisions of the reactants and the probability that a collision will lead to binding determine the kinetics of a signaling reaction. Or in other words, the concentration of the reactants and the rate constant, which includes the summed diffusion speed and the probability that a collision will lead to binding, determine the kinetics.

The concentration of reactants and hence collision frequency are dramatically increased when diffusible space is reduced from three dimensions in the cytosol to two dimensions on the membrane, which has been shown by Kholodenko and coworkers especially for receptor tyrosine kinase (RTK) mediated signaling [17]. Nevertheless, the increase in concentration is accompanied by a reduction in the diffusional mobility of the reactants. Whether dimensionality reduction provides a gain in the reaction rate has been analyzed for extracellular ligands by Axelrod and Wang, and the authors concluded that reaction rates do increase, although the mobility of the reactants is decreased [4].

Particularly, RTK-Ras signaling has been studied in this context. Ras is a GTPase and not only is a major oncogenic signaling protein but also involved in hypertrophic and survival signaling of cardiac myocytes.

Ras interacts with and thereby recruits effectors to the plasma membrane only when activated by guanine nucleotide exchange factors (GEFs). These factors form complexes with adaptor proteins binding to activated RTKs, and activated Ras GTP will recruit and concentrate effectors such as BRaf. BRaf itself is activated in asymmetric dimers containing activator kinase that induces *cis*-autophosphorylation in the activation loop of the receiver kinase [16]. Interaction of BRaf with Ras triggers a conformational change to allow the asymmetric dimerization to occur and enhances BRaf dimerization [34] using the above-mentioned dimensionality reduction. Phosphorylation of CRaf, which is also able to interact with Ras [14], in the N-terminal acidic motif by membrane-associated kinases such as Src [11] or protein kinase C (PKC) [26] allows it to become the activator kinase in asymmetric dimers [9]. PKC is recruited to the plasma membrane by the lipid second messenger diacylglycerol, and most of PKC isoforms are activated by calcium, thus linking CRaf phosphorylation to calcium transients in cardiac myocytes. There are even more direct links to mechanosensation as Src kinase, which to a large extent transmits integrin-mediated signals and which localizes to costameres, can also be viewed as a mechanically induced kinase [45] and which has been shown to phosphorylate p130 Cas upon stretch [40]. Moreover, Ras mutations are a well-known cause of LEOPARD and Noonan syndromes, which are characterized by skin, skeletal, and cardiovascular defects, which include syndromic forms of hypertrophic cardiomyopathy and which are called RASopathies (for a review: Gelb and Tartaglia [13]).

In summary, the ERK1/2 pathway is particularly important in the heart as it integrates both growth and cell survival, depending on the prevailing amount and nature of biomechanical stress [30]. CRaf and its activating kinases are translocated and concentrated on the sarcolemma, which largely increases the kinetics of CRaf aminoterminal phosphorylation and the propagation of signals via the MAPK pathway.

### Taking into account membrane curvature

The above-mentioned concepts do not take into account inhomogeneity in membrane curvature, which may also affect protein association and hence signaling. It is reasonable to assume that in cavities—where high surface to cytosol ratios exist—local resupply via diffusion is limited. Exactly the opposite should happen for protruding membrane folds that have a low local surface to cytosol ratio and where a large diffusible cytosolic pool is available.

Precisely, these effects have recently been studied, and a theory of this concept of locality of surface to volume ratios has been developed, and it was shown that this operates in signaling [37]. Rangamani and coworkers combined (1) reaction kinetics, (2) diffusion, and (3) membrane shape and showed how a transient enhancement of receptor activity after increased ligand binding in curved plasma membrane areas can mediate enhancement of MAPK or Src kinase activity in downstream signaling pathways. In other words, information contained in a cell’s shape can be transformed into different biochemical signaling activities. This effect, of course, is short lived as diffusion will equilibrate ligand-induced inhomogeneity of receptor activity but may well play a more prominent role in dynamic systems such as the heart.

### Applications of the concept of locality of surface to volume ratios

The above-described effect of locality of surface to volume ratios may play a role in endocytosis, where maximized signaling activity of endocytosed RTKs has been observed [43], or it may play a role in mitochondria, where cristae morphology determines assembly and activity of the respiratory chain complexes (for a recent review please, see Schmick and Bastiaens [41]).

This effect also might be important for differentiating embryonic stem cells, where in mesenchymal stem cells, spreading has been shown to give rise to osteocytes, whereas round cells evolved into adipocytes. In this set of experiments, cell shape has been shown, at least to some extent, to determine the activity of the RhoA-ROCK pathway [31].

Local membrane inhomogeneity may also play a role in dynamic organs such as the heart, where cardiac myocytes change their shape frequency dependent and drag or release other cell types resident in the heart such as cardiac fibroblasts. Cardiac fibroblasts are the primary producers of ECM in the heart and cardiac myocyte—cardiac fibroblast communication plays a major role in many cardiovascular diseases such as hypertrophy, hypertension, and coronary heart disease (for a review: Fujiu and Nagai [12]).

Cardiac myocytes and cardiac fibroblasts are highly interspersed in the myocardium with several fibroblasts surrounding a single myocyte. Bidirectional cross talk between these cell types plays important roles in determining cardiac mechanical and electrical function in normal and diseased hearts. Mechanisms involved include the release of paracrine factors such as TGFβ, IL family members, TNFα, VEGF, FGF2, endothelin, and AngII; direct cell–cell interactions via gap junctions and possibly adherence junctions and nanotubes; and interactions with the ECM (for an excellent review: Zhang et al. [46]). VEGF seems to be of particular importance as it can be secreted from injured cardiac myocytes and which, in turn, acts as a paracrine factor to induce myofibroblast proliferation, suggesting that VEGF also contributes to fibrosis in the diseased heart [35]. In addition, the importance of TGFβ for hypertrophic cardiomyopathy and associated fibrotic remodeling processes has recently been demonstrated [44]. The recent observation of gap junctions existing between cardiac fibroblasts and cardiac myocytes, which most likely play a role in the prevention of arrhythmias, is certainly remarkable and will provide new impetus for therapy [3, 32].

Cardiac fibroblasts will, depending on the frequency of the beating heart, periodically elongate and shrink. High frequencies are predicted to increase the probability of a fibroblast being in elongation and thus sensitize the cell to growth factor stimulation.

Local inhomogeneity of the membrane could also explain why some preexcitation syndromes, which are associated with periodic increases in frequency, appear hypertrophic and develop fibrosis [2]—an effect probably related to cardiac fibroblasts, which also have been studied in the report by Rangamani and coworkers [37].

Aside from RTKs such as epidermal growth factor receptor (EGR), which couples to Ras, or Src kinase, and costamere-related signaling, beta adrenergic receptors (βARs) are very important for cardiac physiology as they convey positive inotrope, bathmotrope, lusitrope, and chronotrope signals. One of the oldest and probably best analyzed heart failure models used in physiology is the pacing-induced heart failure model in dogs [10]. While contraction may provide costameres with more diffusional space and thus enhance signaling via these structures in systole, βARs are likely to be equally distributed over the sarcolemma, and therefore, βAR-mediated signaling is more likely to seize, which is exactly what has been observed in pacing-induced heart failure [7].

**Fig. 1:**
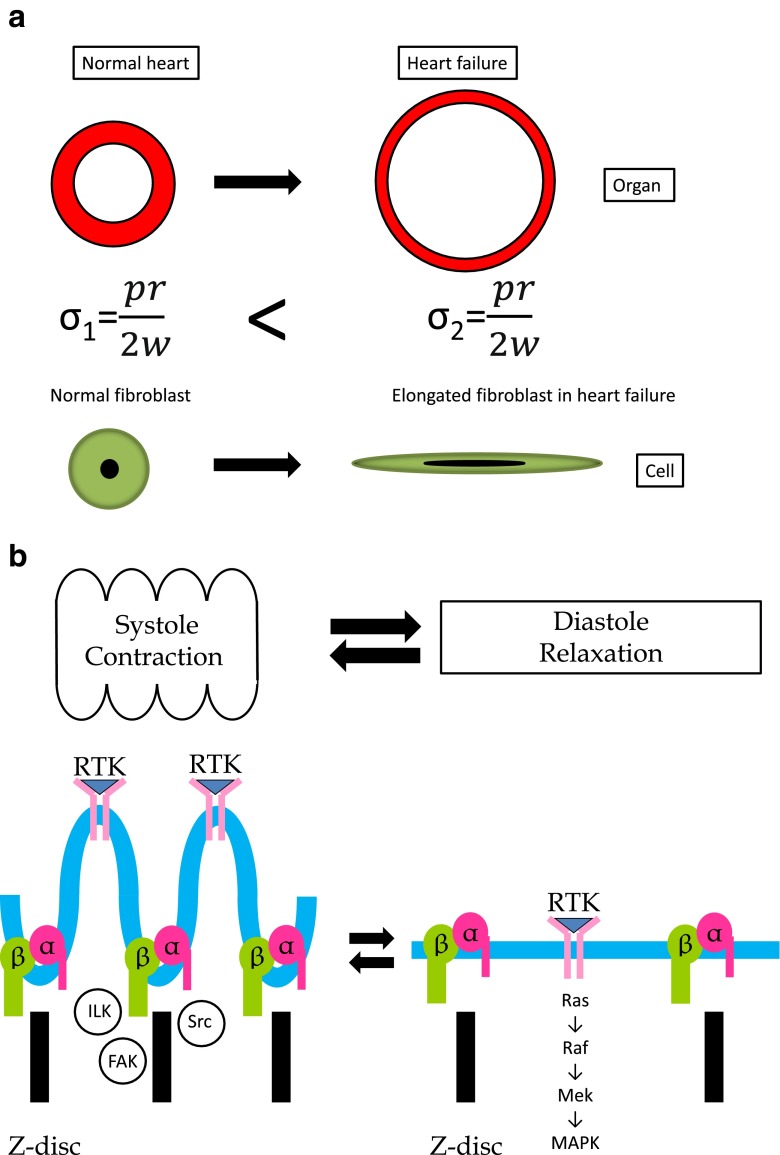
**a** Heart failure is associated with an increase in wall stress as a result of increased cardiac dimensions and loss of wall thickness, which will stretch all myocardial cells, as schematically shown for a cardiac fibroblast. **b** Cardiac myocytes contract and enlarge periodically during systole and diastole, respectively. This will specifically affect cardiomyocyte membrane shapes: During systole costameres are more exposed to the cytosol, which will increase this type of signaling. Parts of the membranes between neighboring Z-discs bulge out and thus increase local membrane to volume ratios, which is predicted to decrease RTK-mediated signaling (and vice versa in diastole)

A βAR-mediated increase in frequency, via changes in membrane shape alone, is expected to activate the Ras–Raf–MAPK pathway and hence to initiate a pro-hypertrophic and pro-apoptotic pathway, while βAR activation is probably attenuated (Fig. 1).

Another consequence of this effect probably can be directly related to cardiac myocytes. Stretched and therefore elongated cardiac myocytes show an increase in MAPK kinase activity such as an upsurge in ERK1/2 phosphorylation, which has been linked to angiotensin II (Ang II) and angiotensin II type 1 receptor (AT_1_R) mediated effects [8]. However, the model of local membrane inhomogeneity predicts that lengthened cardiac myocytes are more prone to receptor mediated and thus AT_1_R-mediated effects. In this context, it is probably important to refer to the report by Zou and coworkers who showed that mechanical stretch alone is sufficient to activate the AT_1_R [47], thus providing a positive feedback mechanism for stretch-mediated effects, at least in cardiac myocytes. Cyclic stretch in vitro in one dimension can be related to volume overload in vivo, and local membrane inhomogeneity may well be related to eccentric types of cardiac hypertrophy.

It is probably worth pointing out that the dynamics of the heart are a good example to study the effects of local membrane inhomogeneity, as the surface areas and volumes of the cells remain constant, which is difficult to prove in the experimental settings used in the original article by Ramagani et al. [37]. However, the effect of cardiac myocyte shape on function has recently been studied in an elegant article by Kuo and coworkers [27]. The authors used in vitro assays to analyze different aspect ratios (i.e., length/width ratios) and found that function clearly depended on the shape of the cardiac myocytes and that shape also determined calcium metabolism.

## Summary

In a simplified model, one can assume that a convex membrane fosters ligand/receptor interaction and hence signaling. Therefore an elongated, elliptical cell will increase its signaling activity via decreasing local membrane to volume ratios. These effects probably can be observed in the myocardium, where contracting myocytes periodically elongate cardiac fibroblasts and hence increase their sensitivity to receptor-mediated effects and which leads to hypertrophy in these cells. An increase in frequency will increase the probability of a cardiac fibroblast being elongated, being activated, and being able to cause cardiac myocyte hypertrophy via cross talk. Or in other words, cardiac fibroblasts will elongate in response to stretch caused by contracting cardiac myocytes which, in turn, will cause hypertrophy (first in cardiac fibroblasts and via cross talk secondarily in cardiac myocytes).

The situation in cardiac myocytes itself probably is more complex: Contraction will expose costameres to the cytoplasm and hence increase this type of signaling (for example, integrin-mediated signaling such as Src kinase, focal adhesion kinase, and integrin-linked kinase [25]). However, the membrane parts between costameres probably are bulging outward, which will increase membrane to cytosol ratios and hence decrease signaling. Higher frequencies will result in a net increase of costamere-related signaling and thus can contribute to the development of (a certain type of) cardiac myocyte hypertrophy and heart failure [10]. The development of heart failure in other models, such the ones whereby an increase in cardiac preload or volume stretches cardiac myocytes and noncardiac myocytes, and which cause eccentric hypertrophy, probably can be better explained by using the concept of local membrane inhomogeneity.

Another aspect of this model is that every hypertrophy will lead to a decrease of surface to volume ratios which increases signaling activity, at least in theory. In the heart, this is expected to cause diastolic defects, decrease the ability of the heart to increase its frequency, and hence may lead to a termination of costamere-related hypertrophic stimuli. Thus, hypertrophy can be regarded as part of a negative feedback loop aimed to equilibrate various membrane-mediated signaling pathways. The effects of local membrane inhomogeneity offer a novel molecular mechanism to explain various types of cardiac hypertrophy, which so far largely has been seen as a response to normalize wall stress.

Although the concept of locality of surface to volume ratios is attractive, it is still very general and to a large extent based on theoretical considerations which depend on specific reaction kinetics and diffusion probabilities and which may differ from receptor to receptor, ligand to ligand, and cell-type to cell-type. In addition, when introducing the concepts of hypertrophy and atrophy, time and adaptation to the change in conditions have to be considered. Additional experimental proof is needed, particularly in the cardiovascular system. Nevertheless, this effect could be important for the heart.

## Conclusion

Mechanosensation is a fundamental process and present in every living cell with particular importance for a dynamic organ such as the heart. Many different mechanosensory mechanisms are at play in cardiac myocytes and include stretch activated (or inactivated) channels, cytoskeletal components or structures such as the sarcomeric Z-disc, or actomyosin interaction per se.

Recently, a novel mechanism has been described whereby cell shape directly translates into signaling and which might be of importance particularly for the heart where cells during systole and diastole continuously reshape membranes. The concept of locality of surface to volume ratios was demonstrated to affect membrane-mediated signaling and may help to explain the remarkable plasticity of the myocardium in response to biomechanical stress.
